# Improving Radiation Response in Glioblastoma Using ECO/siRNA Nanoparticles Targeting DNA Damage Repair

**DOI:** 10.3390/cancers12113260

**Published:** 2020-11-04

**Authors:** Jennifer A. Lee, Nadia Ayat, Zhanhu Sun, Philip J. Tofilon, Zheng-Rong Lu, Kevin Camphausen

**Affiliations:** 1 Radiation Oncology Branch, National Cancer Institute, National Institutes of Health, Bethesda, MD 20892, USA; philip.tofilon@nih.gov (P.J.T.); camphauk@mail.nih.gov (K.C.); 2 Department of Biomedical Engineering, Case Western Reserve University, Cleveland, OH 44140, USA; nra21@case.edu (N.A.); zxs219@case.edu (Z.S.); zxl125@case.edu (Z.-R.L.)

**Keywords:** nanoparticles, siRNA, glioblastoma, radiation, DNA damage repair, gene silencing

## Abstract

**Simple Summary:**

Glioblastoma (GBM) is the most common form of brain cancer and among the most lethal of human cancers. Radiation therapy is a mainstay in the standard of care for GBM, killing tumor cells by creating DNA damage. Inhibiting DNA damage repair (DDR) proteins enhances radiation therapy by not allowing tumor cells to repair the DNA damage caused by radiation. The aim of our study was to investigate whether the novel nanoparticle material, ECO, could be used to deliver small interfering RNA (siRNA) to GBM tumor cells and temporarily reduce the production of DDR proteins to improve radiation therapy outcomes. SiRNAs can be designed to target an innumerable number of genes and with the right delivery vehicle can be used in a variety of disease settings. Our work provides support for the use of the novel ECO material for delivery of siRNA in GBM.

**Abstract:**

Radiation therapy is a mainstay in the standard of care for glioblastoma (GBM), thus inhibiting the DNA damage response (DDR) is a major strategy to improve radiation response and therapeutic outcomes. Small interfering RNA (siRNA) therapy holds immeasurable potential for the treatment of GBM, however delivery of the siRNA payload remains the largest obstacle for clinical implementation. Here we demonstrate the effectiveness of the novel nanomaterial, ECO (1-aminoethylimino[bis(N-oleoylcysteinylaminoethyl) propionamide]), to deliver siRNA targeting DDR proteins ataxia telangiectasia mutated and DNA-dependent protein kinase (DNApk-cs) for the radiosensitzation of GBM in vitro and in vivo. ECO nanoparticles (NPs) were shown to efficiently deliver siRNA and silence target protein expression in glioma (U251) and glioma stem cell lines (NSC11, GBMJ1). Importantly, ECO NPs displayed no cytotoxicity and minimal silencing of genes in normal astrocytes. Treatment with ECO/siRNA NPs and radiation resulted in the prolonged presence of γH2AX foci, indicators of DNA damage, and increased radiosensitivity in all tumor cell lines. In vivo, intratumoral injection of ECO/siDNApk-cs NPs with radiation resulted in a significant increase in survival compared with injection of NPs alone. These data suggest the ECO nanomaterial can effectively deliver siRNA to more selectively target and radiosensitize tumor cells to improve therapeutic outcomes in GBM.

## 1. Introduction

Glioblastoma (GBM) is the most common and lethal primary brain tumor. Despite advances in precision medicine and targeted therapies, the standard of care for GBM continues to be surgical resection followed by concomitant radiation and temozolomide. With this treatment regimen the overall survival for patients with GBM is a sobering ~14 months with a 5-year survival of ~5% [1,2]. Because radiation has been a mainstay in the treatment of GBM, much of the research to improve patient outcomes has focused on finding therapies to bolster the effects of radiation by radiosensitizing GBM tumor cells. A primary method for radiosensitization of tumor cells is to inhibit the repair of DNA double-strand breaks caused by radiation, leading to cell death [3,4,5]. Two proteins commonly targeted for radiosensitization include ataxia telangiectasia mutated (ATM) and DNA-dependent protein kinase (DNApk), which are involved in the DNA repair processes homologous recombination and non-homologous end joining, respectively. Drugs blocking ATM and DNApk have shown to radiosensitize GBM cells in preclinical settings, but those successes have yet to translate into the clinic [6,7,8]. Major obstacles to the use of these drugs include off-target effects and toxicity that limit the ability to deliver doses across the blood brain barrier that produce sufficient radiosensitization for increases in overall survival [7,9].

An alternative strategy to specifically inhibit a therapeutic target is the use of small interfering RNA (siRNA), which are short, double-stranded RNA designed to mark complementary mRNA for degradation and downregulate the expression of a gene [10]. RNA interference (RNAi) technologies are highly specific and efficient in comparison to drug therapeutics, and further can affect proteins that are difficult to target via drugs or antibodies [11,12]. However, at present only two RNA-based therapies have been FDA approved, Onpattro^®^ (patisiran) and Givlaari^®^ (givosiran) (Alnylam Pharmaceuticals), which treat polyneuropathy of hereditary TTR-mediated amyloidosis and acute hepatic porphyria, respectively [13,14,15,16]. While siRNAs are easily synthesized, the lack of RNAi use in clinical applications can be largely attributed to difficulties in the packaging and delivery of the siRNA payload [17,18,19].

Nanoparticles are of significant interest for drug delivery, especially for the implementation of siRNA therapies. These particles are often composed of lipids, polymers, or are found in inorganic forms such as carbon or gold nanoparticles [20,21]. Indeed, both the aforementioned Onpattro^®^ and Givlaari^®^ utilize lipid nanoparticles for siRNA delivery. Lipid-based nanoparticles are easily customizable to target specific tumor types, avoid degradation and clearance, and efficiently carry siRNA into the desired cell, making them an attractive carrier for siRNA therapies [22]. For example, functionalization can include components such as polyethylene glycol (PEG), a hydrophilic polymer, to shield nanoparticles from clearance by the reticuloendothelial system, and the peptide Arg-Gly-Asp (RGD) to selectively target integrins like α_v_β_3_ that are overexpressed in GBM [23,24,25].

1-aminoethylimino[bis(N-oleoylcysteinylaminoethyl) propionamide] (ECO) is a pH-sensitive, amino lipid-based carrier that has been successfully used to deliver siRNA in preclinical studies of breast cancer [26,27,28]. Moreover, this nanocarrier has been shown to have a stable and prolonged shelf-life when frozen, making it a more attractive candidate for clinical use [20]. In this study we investigated the ability of the pH-sensitive ECO nanocarrier to deliver siRNA for the radiosensitization of GBM and GBM stem-like cell lines in vitro and in vivo. ECO nanoparticles were functionalized with RGD-PEG and loaded with siRNA targeting ATM, the catalytic subunit of DNApk (DNApk-cs), or their combination. The effects of ECO nanoparticle delivery and radiation was evaluated on cell viability, protein expression, DNA damage repair, and clonogenicity in normal astrocytes, a GBM cell line (U251), and two glioma stem-like cell lines (NSC11 and GBMJ1) in vitro. Further, to assess the in vivo effect of nanoparticles in combination with radiation on survival, RGD-PEG labeled ECO nanoparticles carrying siDNApk-cs were intratumorally injected and combined with fractionated radiation in an orthotopic U251 mouse model. Taken together, our studies show that the novel ECO nanomaterial can be utilized for the delivery of siRNA to improve radiation response in GBM.

## 2. Results

### 2.1. ECO Nanoparticles Are Not Toxic to Normal Astrocytes and Deliver siRNA to GBM Cells and GSCs

**Figure 1:**
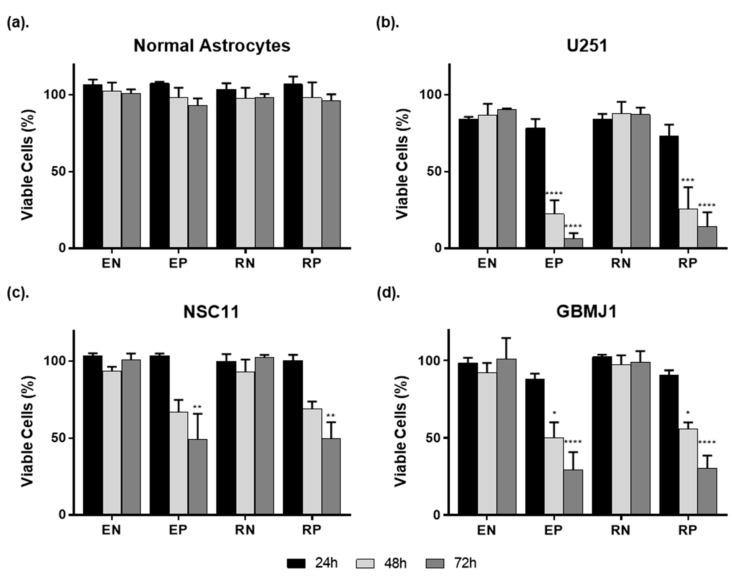
Cell viability over time during exposure to unlabeled and Arg-Gly-Asp and polyethylene glycol (RGD-PEG) labeled ECO nanoparticles (NPs). (**a**) Normal astrocytes, (**b**) U251 GBM cells, and GBM stem-like cell lines (**c**) NSC11 and (**d**) GBMJ1 were evaluated. Cells receiving no NP treatment were used as control for each day (100% viability). Values are shown as the mean ± SEM of 3 independent experiments. (Significance compared to negative siRNA controls and calculated by ANOVA with Tukey post-analysis: * *p* < 0.5, ** *p* < 0.01, *** *p* < 0.001**** *p* < 0.0001); (E—unlabeled NPs, R—RGD-PEG labeled NPs, N—negative control siRNA, P—positive control cell kill siRNA).

We first investigated the cytotoxicity of unlabeled (E) and RGD-PEG (R) labeled ECO nanoparticles (NPs) on normal astrocytes (NAs), glioblastoma (GBM) tumor cells, and glioma stem-like cells (GSCs) using an ATP-based cell viability assay (Figure 1). The delivery of non-specific siRNA was used to evaluate the toxicity of NPs alone. Positive cell-kill siRNA was used to evaluate the effectiveness of siRNA delivery. All groups were compared to cells receiving no NP treatment. NPs were plated and viability assessed at 24, 48, and 72 h thereafter with NPs left on cells for the duration of the experiment.

There was no difference between unlabeled and labeled particles on NA viability irrespective of the siRNA being delivered. The lack of cell kill observed following delivery of positive control siRNA may indicate a reduced capacity for ECO NPs to deliver siRNA to NAs. In contrast, the ECO NPs delivered siRNA effectively to GBM and GSC lines. In the adherent U251 GBM cell line, exposure to either labeled (RN) or unlabeled (EN) NPs carrying negative control siRNA resulted in a non-significant decrease in cell viability by 10–15% compared to cells not receiving NP treatment. NP delivery of positive cell kill siRNA resulted in a significant decrease in cell viability at 48 and 72 h, indicating successful uptake and processing of the encapsulated siRNA. At 72 h U251 cells demonstrated cell kill down to ~6% and ~14% for unlabeled (EP) and labeled particles (RP), respectively.

Because the treatment resistance of GBM is thought to be attributed to the presence of GSCs, NPs were plated on two different GSC lines. NPs alone showed no significant effect on NSC11 or GBMJ1 viability. Much like the U251 cell line, the delivery of positive cell kill siRNA resulted in a gradual increase in cell death by 48 h, with significant cell kill at 72 h of ~50% for NSC11 and ~30% for GBMJ1 cells. Because the use of RGD-PEG has been shown to prove beneficial for targeting and clearance evasion in vivo, labeled nanoparticles were used for the rest of the study [23,24,25].

### 2.2. ECO Nanoparticle Delivery of siRNA Results in Greater Gene Silencing in GBM Tumor Cells Compared to Normal Astrocytes

**Figure 2:**
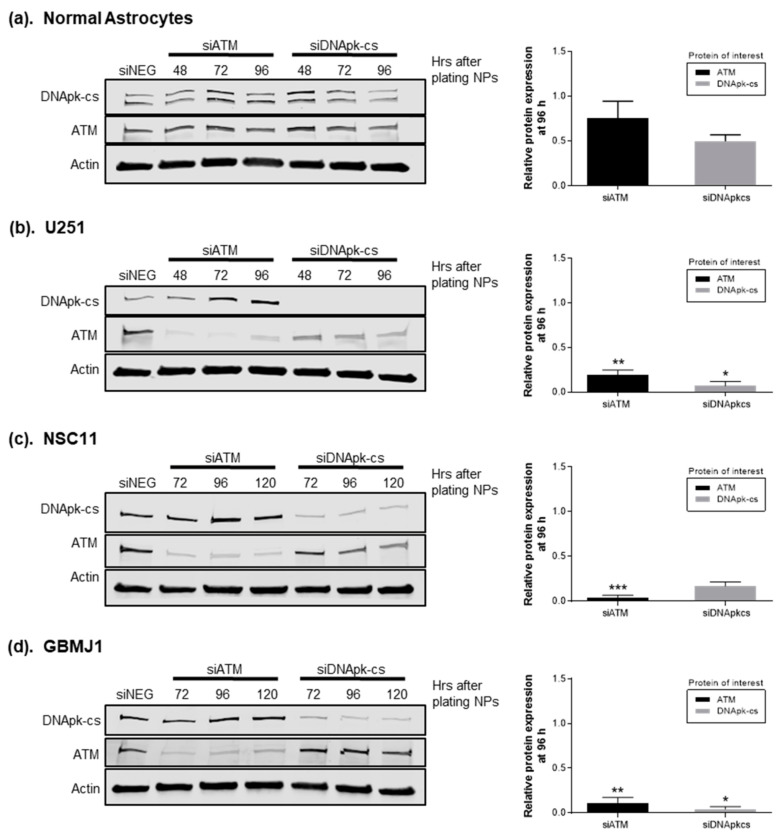
Silencing of target genes via RGD-PEG labeled ECO nanoparticles (NPs) carrying ATM and DNApk-cs siRNA. The relative protein expression for ATM and DNApk-cs at 96 h after plating NPs compared to cells receiving control siRNA is represented (mean ± SEM of 3 independent experiments). (**a**) Normal astrocytes showed limited silencing after exposure to NPs compared to tumor cell lines (**b**–**d**). Significance is compared to the protein expression values of normal astrocytes and determined by ANOVA with Tukey post-analysis (* *p* < 0.5, ** *p* < 0.01, *** *p* < 0.001).

Ataxia telangiectasia mutated (ATM) and DNA dependent protein kinase (DNApk) are major components of the DNA repair processes homologous recombination and non-homologous enjoining, respectively [29,30]. As these proteins have been shown as viable targets for radiosensitization, both were chosen to demonstrate ECO NP delivery of siRNA. Western blotting was used to show the degree and time course of gene silencing in NAs, GBM, and GSC lines following delivery of siRNA with RGD-PEG labeled ECO NPs. NPs carrying 40 nM of siRNA targeting ATM or the catalytic subunit of DNApk (DNApk-cs) were plated on cells for 24 h, after which the NP-containing media were removed, cells rinsed with phosphate buffered saline (PBS), and fresh media added. Cells were collected at timepoints between 48 and 120 h after plating of NPs. Figure 2 shows representative protein blots for each cell line tested and the relative protein expression of ATM and DNApk-cs at 96 h after plating of NPs compared to cells receiving control siRNA. Similar to our cell viability results, NAs (Figure 2a) showed the least amount of protein silencing down to 0.75 ± 0.19 fold expression of ATM and 0.50 ± 0.07 fold expression of DNApk-cs. The NPs induced a much greater degree of silencing in tumor cell lines (Figure 2b–d), with ATM expression reduced to 0.19 ± 0.06, 0.04 ± 0.02, and 0.11 ± 0.06 and DNApk-cs expression reduced to 0.07 ± 0.05, 0.17 ± 0.05, 0.04 ± 0.03, in U251, NSC11, and GBMJ1, respectively. These results show that delivery of siRNA by RGD-PEG labeled ECO NPs results in effective silencing and a more significant impact on GBM tumor cells compared to NAs.

### 2.3. GBM and GSC Lines Show Radiosensitization after Treatment with RGD-PEG Coated ECO NPs Carrying DNA Repair siRNAs

**Figure 3:**
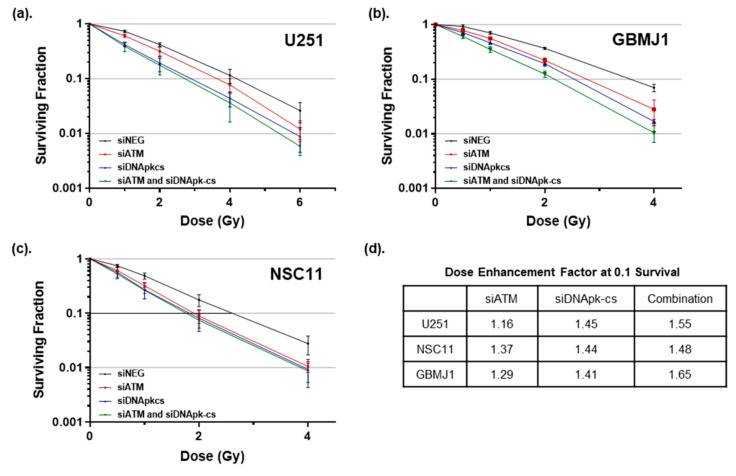
Clonogenic cell survival and corresponding dose enhancement factors in glioma ((**a**) U251) and glioma stem cells ((**b**) GBMJ1, (**c**) NSC11) after treatment with nanoparticles targeting ATM, DNApk-cs, and a combination of the two targets. Clonogenic curves are shown as the mean ± SEM of 3 independent experiments. Dose enhancement factors (**d**) were calculated at a surviving fraction of 0.1.

The clonogenic cell survival assay is the gold standard for assessing the effect of therapeutics on radiosensitivity in vitro (Figure 3) [31]. As with previous immunoblot experiments, cells were treated with RGD-PEG coated ECO NPs carrying ATM siRNA (40 nM), DNApk-cs siRNA (40 nM), or a combination of the two (20 nM each) for 24 h at which time the NP-containing medium was removed and fresh media added. Using the timepoint of maximum silencing for U251 cells, cells were irradiated (0.5–6 Gy) 72 h after NP removal. The combination treatment resulted in the greatest increase in dose enhancement factor (DEF), and thus radiosensitivity, across all cell lines. For the adherent GBM cell line U251, the NP treatment resulted in DEFs of 1.16, 1.45, and 1.55 for siATM, siDNApk-cs, and their combination, respectively. The DEFs for the GSC lines were 1.37, 1.44, and 1.48 in NSC11, and 1.29, 1.41, and 1.65 in GBMJ1 for the siATM, siDNApk-cs, and combination treatment. Though all three siRNA payloads resulted in similar DEFs for NSC11, targeting ATM was the least effective strategy for all cell lines. Targeting DNApk-cs resulted in significant radiosensitization for all cell lines, however the combination of siRNA targeting both ATM and DNApk-cs resulted in the largest increase. Overall, the RGD-PEG labeled ECO nanoparticles again demonstrated effective delivery of siRNA to GBM and GSC lines, and more importantly demonstrated the capability to carry different siRNA in the same payload to inhibit multiple targets.

### 2.4. ECO NP Delivery of siRNA Inhibits DNA Double Strand Break Repair

**Figure 4:**
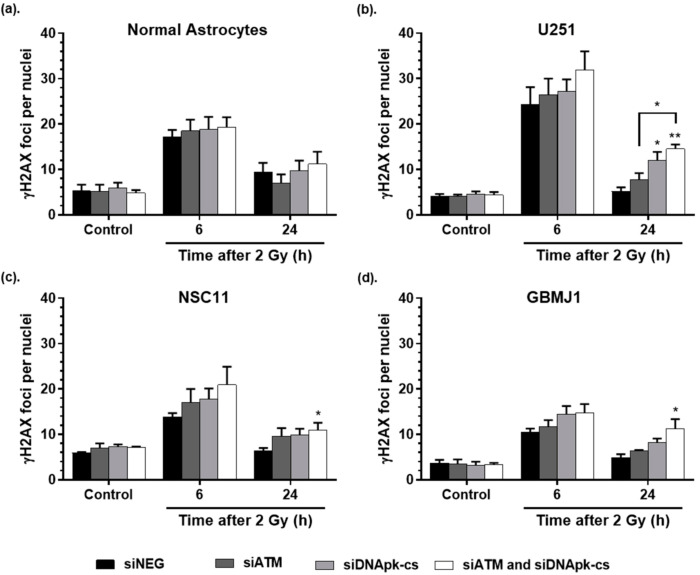
Influence of ECO nanoparticle delivery of siRNA on gH2AX foci formation and repair in (**a**) normal astrocytes, (**b**) glioma, and (**c**,**d**) glioma stem cell lines. Cells receiving no radiation were used as a control. Foci were counted in 50 nuclei per treatment, per experiment. Values are represented as the mean ± SEM of 3 independent experiments. (Significance in comparison to siNEG at 24 h unless indicated otherwise according to ANOVA testing with Tukey post-analysis (* *p* < 0.05, ** *p* < 0.01).

The ability to repair radiation-induced DNA double strand breaks (DSBs) following treatment with RGD-PEG labeled ECO NPs was evaluated using the γH2AX foci assay (Figure 4). As with the clonogenic survival assay, cells were incubated with RGD-PEG coated ECO NPs for 24 h, NPs removed, and fresh media added. At 72 h after removal of NPs, cells were irradiated (2 Gy) and fixed at the indicated timepoints after irradiation. Normal astrocytes (Figure 4a) showed no difference in the number of foci at 6 or 24 h after radiation irrespective of treatment used, indicating that any silencing observed in previous protein expression experiments had no effect on DNA DSB repair. Interestingly, for tumor cell lines at 24 h after radiation, only the combination siRNA treatment resulted in a significant increase in remaining foci compared to controls for each tumor cell line. With respect to delivery of each siRNA alone, only delivery of DNApk-cs siRNA resulted in a significant increase in foci for the U251 cell line (Figure 4b), and a greater, but non-significant increase for the GSC lines (Figure 4c,d). Overall, these data demonstrate that effective delivery of siRNA by RGD-PEG labeled ECO NPs can interfere with the repair of radiation-induced DSBs, with siDNApk-cs or the combination of ATM and DNApk-cs having the greatest impact on foci repair.

### 2.5. Effect of Intratumoral Injection of RGD-PEG Labeled ECO NPs on Radiation Response in an U251 Orthotopic Model of GBM

**Figure 5:**
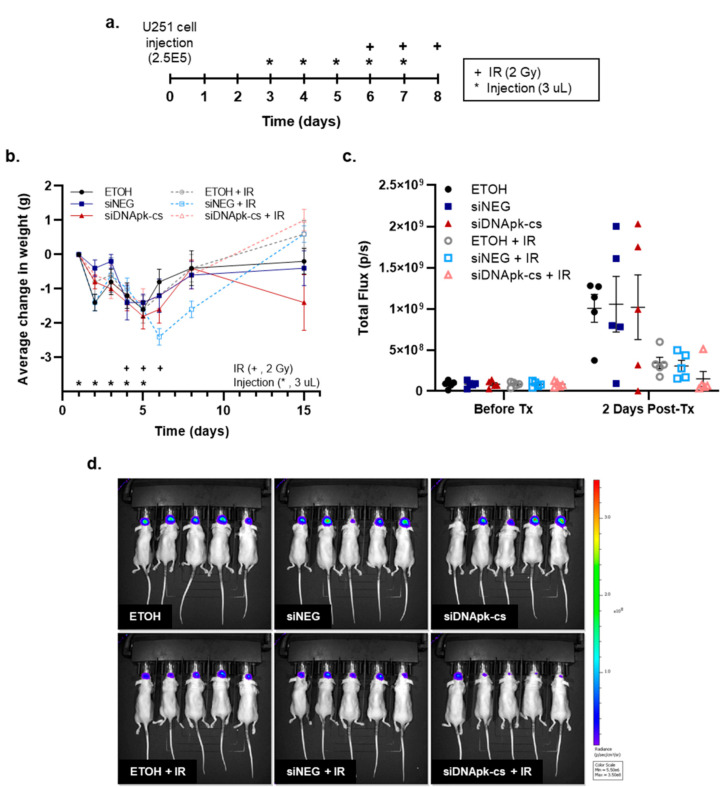
In vivo evaluation of treatment with intratumorally injected RGD-PEG labeled ECO nanoparticles. (**a**) Experimental schedule. (**b**) Average change in animal weight with respect to the first day of injection (mean ± SEM). (**c**) Average total flux of mice before and 2 days after treatment (Tx) (mean ± SEM). (**d**) Image of bioluminescence measurements for all mice 2 days post-Tx.

To show the use of RGD-PEG labeled ECO NPs to deliver siRNA in vivo we performed intratumoral injections of ECO NPs in an orthotopic, xenograft mouse model of GBM using U251 cells. We chose to use siRNA targeting DNApk-cs alone due to the significant radiosensitization observed in the U251 cell line in vitro. A guide screw was surgically implanted in the skulls of mice and used as a port to inject tumor cells and NP treatments. Because the NP material was dissolved in ethanol (ETOH), ETOH (7%) was used as a control, equivalent to that found in NP injections. All mice were injected intratumorally for 5 consecutive days. Mice receiving radiation were irradiated for 3 consecutive days starting on the fourth day of injection (Figure 5a; * intratumoral injections (3 µL), + irradiation (2 Gy)). Starting radiation on the fourth day of injections allowed time for the siRNA to inhibit protein expression in tumor cells. Figure 5b illustrates the average change in animal weight with respect to their starting weight on day 1 of injections. All mice experienced a decrease in weight presumably due to the daily intratumoral injections and handling of the animals. There was no significant difference between mice receiving ETOH and mice receiving NP injections, indicating that the intratumoral injection itself was the cause of the weight drop and not the material being injected.

Bioluminescence (BLI) measurements (Figure 5c) served as a biomarker of tumor progression following cessation of treatment. Mice were imaged 2 days following the last dose of radiation. While irradiated mice overall had lower BLI values compared to non-irradiated mice, mice treated with NPs carrying DNApk-cs siRNA and radiation showed the largest decrease in BLI in comparison to any other control or treatment group. This drop in BLI is a rough indicator of cell kill from the treatment. BLI images for each experimental group 2 days post treatment are shown in Figure 5d. 

**Figure 6:**
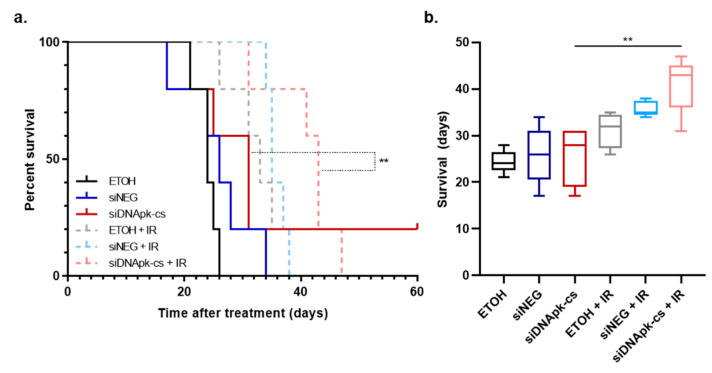
Effect of ECO nanoparticle (NP) delivery of siDNApk-cs on the radioresponse of U251 orthotopic xenografts. (**a**) Kaplan–Meier survival curves showing the effect of siDNApk-cs delivery by RGD-PEG labeled ECO NPs. Significance calculated using log-rank analysis (** *p* < 0.01). One mouse in the siDNApk-cs alone and ethanol (ETOH) + IR groups displayed complete tumor regression. Mice were monitored until the onset of morbidity. (**b**) Median survival of experimental groups. Significance calculated using ANOVA with Tukey post-analysis (** *p* < 0.01).

Though we observed a decrease in BLI signal soon after treatment, the question remained whether this would translate into a survival benefit (Figure 6a). Mice receiving control injections of ETOH had a median survival of 24 days. The NP delivery of negative control siRNA or siDNApk-cs resulted in non-significant increases in survival by 2 and 7 days. The addition of radiation to injections of ETOH and ECO NPs carrying siNEG increased survival by 9 and 11 days, respectively. Like our BLI imaging predicted, mice receiving treatment with RGD-PEG labeled ECO NPs carrying DNApk-cs siRNA resulted in the longest survival with an increase in median survival of 19 days beyond control mice receiving ETOH only. This increase in survival was significantly greater than the delivery of siDNApk-cs alone (Figure 6b) and demonstrated that the delivery of siRNA targeting DNApk-cs was successful in producing a radiosensitizing effect, even with limitations associated with intratumoral injections.

## 3. Discussions

RNA interference therapy has growing potential for the treatment of GBM and other cancers due to the ability to target innumerable molecular targets and a lower toxicity compared to conventional chemotherapeutics [11,12]. However, the largest obstacle to the implementation of this therapy continues to be the delivery of the siRNA cargo. 1-aminoethylimino[bis(N-oleoylcysteinyl-aminoethyl)propionamide] (ECO) is a stable, biocompatible nanoparticle (NP) material that has previously been shown to have significant promise in the breast cancer setting. In these studies, intravenously injected Arg-Gly-Asp and polyethylene glycol (RGD-PEG) labeled ECO NPs delivered siRNA targeting β3 integrin, DANCR, and eIF4F expression to reduce metastasis of triple-negative breast cancer and chemotherapeutic resistance [26,27,28]. Importantly, the ECO material has been developed to maintain stability when frozen after complexation with siRNA, making the ECO nanocarrier an excellent candidate for siRNA therapy in the clinic [20]. Particle delivery of siRNA to treat GBM and modulate its response to temozolomide has been demonstrated in the preclinical setting, however these studies have focused on different gene targets, subcutaneous tumors, or higher doses of siRNA [32,33,34,35,36]. Further, very few studies have been performed evaluating siRNA therapy in combination with radiation for the treatment of GBM [37]. In our study, we used the novel RGD-PEG ECO carrier to deliver siRNA targeting DNA damage repair proteins and radiosensitize GBM in vitro and in vivo, demonstrating the promising potential for siRNA therapy using the ECO nanomaterial in combination with radiation.

Targeting DNA damage repair pathways is a prevalent strategy to improve therapeutic outcomes. We thus evaluated ECO delivery of siRNA targeting ATM and DNApk-cs, two major targets for the radiosensitization of GBM and other cancers [8,38,39]. The RGD-PEG labeled ECO NPs were able to effectively deliver each siRNA and silence target genes in an adherent GBM cell line (U251) as well as GSC lines (NSC11, GBMJ1) (Figure 2). This effective silencing was observed as soon as 48 h after plating NPs and for at least up to 120 h after 24 h of exposure to particles, demonstrating the enduring effect of ECO NP delivery of siRNA. More importantly, we observed no toxicity (Figure 1) and a diminished capacity to silence genes (Figure 2) in normal astrocytes. A potential explanation for these observations may be the inherent physical characteristics of primary astrocytes, which are known to be difficult to transfect [40,41] and like normal cells undergo less cell division and possess less endocytic activity in comparison to tumor cells [42].

It is becoming increasingly evident that in order to improve radiation therapy outcomes in GBM, a multi-targeted approach may be necessary [43,44]. The potential to deliver multiple siRNA has been demonstrated by Kozielski et al., who investigated the nanoparticle delivery of five anti-GBM siRNA to induce cell death and reduce migration in vitro and reduce tumor burden in vivo [45]. In our study we used the ECO nanomaterial to deliver multiple siRNA targeting DNA repair in one payload. ATM and DNApk-cs function primarily in complementary DNA damage repair pathways, homologous recombination and non-homologous end joining, respectively, and as such we hypothesized that targeting both pathways simultaneously would result in greater radiosensitization than targeting either pathway alone. Indeed, the delivery of smaller concentrations of both ATM and DNApk-cs siRNA resulted in the greatest inhibition of DNA damage repair and radiosensitization for all tumor cell lines tested (Figure 3 and Figure 4). Targeting multiple pathways always carries the potential for additional toxicity, however our study showed that while the combination siRNA treatment significantly inhibited the repair of radiation-induced DNA damage in all tumor cell lines tested, normal astrocytes showed no significant difference in DNA damage repair for any siRNA treatment delivered. Based on these findings, ECO nanoparticles show vast potential to increase the therapeutic index of radiation by sparing normal tissues and radiosensitizing tumor tissues. Further, these results highlight the importance of a stable and safe nanocarrier material like ECO, where a single nanoparticle can carry siRNA targeting multiple genes without the increased toxicity that can be seen with combining drug therapies. 

Systemic drug delivery on its own is extremely complex, and delivery to GBM and other brain maladies is made even more complicated by the presence of the blood brain barrier, which inhibits 95% of molecules from crossing into the brain [46]. To circumvent this obstacle while also reducing toxicity associated with systemic delivery, we evaluated intratumoral injection of ECO NPs via guide screw in an orthotopic, U251 xenograft model [47]. The use of intratumoral injections in the preclinical setting has become a more relevant model with respect to clinical comparison due to improvements in convection enhanced delivery (CED), which uses continuous positive pressure to infuse drugs and therapies directly into a brain tumor [48]. As such, there are a number of clinical trials investigating the use of CED to deliver therapies alone as well as in combination with chemotherapy and radiation [49]. Because U251 cells showed significant radiosensitization following treatment with ECO NPs carrying siDNApk-cs alone we chose the single siRNA treatment for in vivo evaluation. We injected 3 µL (10 pmol) of ECO NPs and siRNA daily for 5 consecutive days. To give time for delivery and action of the siRNA, we administered 2 Gy of radiation on days 4, 5, and 6 for a total dose of 6 Gy (Figure 5). By combining ECO NP delivery of siDNApk-cs with radiation the survival was significantly increased compared to NP delivery alone (Figure 6). Similarly, Kievit et al. used iron oxide nanoparticles functionalized with chlorotoxin to deliver siRNA targeting APE1 and show radiosensitization of GBM. In this study nanoparticles were delivered systemically for 5 consecutive days (20 µg of siRNA per day) with each injection followed by 2 Gy of radiation 24 h after injection for a total of 10 Gy [37]. Regardless of route of administration it will be imperative to investigate the timing and dosage necessary for effective translation of siRNA therapies with radiation in the clinic. In addition, exploration into the use of siRNA targeting alternative gene targets could yield more effective radiosensitization. Lastly, a direct comparison of the effectiveness and toxicities associated with systemic and CED delivery of siRNA therapies will be needed.

## 4. Materials and Methods 

### 4.1. Nanoparticle Synthesis

The cationic lipid ECO (MW = 1023) and the targeting ligand RGD-PEG (PEG, 3.4k, Creative PEGworks, Durham, NC, USA) were synthesized as previously described [28,29]. All purchased siRNAs were dissolved in nuclease free water to a concentration of 25 μmol/L. RGD-PEG was dissolved in nuclease free water to a concentration of 0.625 mmol/L. ECO was dissolved in 100% ethanol to a stock concentration of 5 mmol/L for in vitro experiments, and 50 mmol/L for in vivo experiments. Nanoparticles were formulated such that the ratio of protonable amines on ECO to the number of phosphates on the siRNA duplex (N/P ratio) equaled 10. Targeted nanoparticle formulations were prepared by first mixing RGD-PEG (2.5 mol%) with ECO in nuclease free water under gentle agitation for 30 min. Immediately afterwards, complexation with siRNAs for thirty minutes formed stable RGD-PEG-ECO/siRNA nanoparticles. The concentration of siRNA used is indicated for each experiment.

### 4.2. Cell Lines

The U251 human GBM cell line (Division of Cancer Treatment and Diagnosis Tumor Repository, NCI) was cultured in Dulbecco’s modified eagle media (DMEM) supplemented with 10% fetal bovine serum. Neurosphere cultures generated from human GBM surgical specimens were used to represent glioma stem-like cells (GSCs). The NSC11 cell line was kindly provided by Dr. Frederick Lang (MD Anderson Cancer Center) [50] and the GBMJ1 cell line was generated at Moffit Cancer Center [51]. GSCs were cultured as neurospheres in DMEM/F-12, supplemented with B27, and human recombinant bFGF (50 ng/mL) and EGF (50 ng/mL). Normal astrocytes were grown in astrocyte medium (ScienCell, 2% FBS). All cells were maintained at 37 °C in 5% CO_2_. All cell lines have been authenticated (Indexx Laboratories) and checked for mycoplasma contamination by PCR analysis. For radiation experiments a 320 kV X-ray machine (Precision X-Ray Inc., North Branford, CT, USA) with 2.0 mm aluminum filtration (300 kV peak; 10 mA) and dose rate of 2.3 Gy/minute was used.

### 4.3. siRNA

SiRNA was purchased from Qiagen targeting the catalytic subunit of DNApk (DNApk-cs) (Hs_PRKDC_5 sense: 5′-CGUGUAUUACAGAAGGAAATT-3′, antisense: 5′-UUUCCUUCUGUAAUACACGAG-3′; Hs_PRKDC_6 sense: 5′-CGGCUAACUCGCCAGUUUATT-3′, antisense: 3′-AAGCCGAUUGAGCGGUCAAAU-5′; Hs_PRKDC_8 sense: 5′-CCCUGUUGACAGUACUUUATT-3′, antisense: 5′-UAAAGUACUGUCAACAGGGTC-3′;) and ATM (Hs_ATM_5 sense: 5′-GGCUAUUCAGUGUGCGAGATT-3′, antisense: 3′-TTCCGAUAAGUCACACGCUCU-5′, Hs_ATM_8 sense: 5′-CCAUGAGUCUAGUACUUAATT-3′, antisense: 5′-UUAAGUACUAGACUCAUGGTT-3′; Hs_ATM_9 sense: 5′-GGCUUAUACGCGCAGUGUATT-3′, antisense: 5′-UACACUGCGCGUAUAAGCCAA-3′; Hs_ATM_12 sense: 5′-CCUGUUUGUUAGUUUAUUATT-3′, antisense: 5′-UAAUAAACUAACAAACAGGTG-3′). Hs_PRKDC_6 and Hs_ATM_5 were chosen for use in this study (Figure S1). The AllStars negative control siRNA was used as a non-silencing control and AllStars Hs Cell Death Control siRNA was used as a positive cell-kill silencing control (Qiagen, Hilden, Germany).

### 4.4. In Vitro Cell Viability

Cell viability following nanoparticle administration was assessed through ATP quantification using the CellTiter-Glo^®^ luminescent assay (Promega, Madison, WI, USA). Naked ECO NPs (E) were compared to NPs coated with RGD-PEG (R). Untreated cells were compared to cells receiving non-silencing negative control siRNA or a positive cell kill siRNA. Cells were seeded in white, opaque 96-well plates and allowed to attach overnight (normal astrocytes 4000 cells/well, U251 4000 cells/well, NSC11 and GBMJ1 7000 cells/well). NSC11 and GBMJ1 cell lines were seeded in plates coated with poly-l-lysine. A total of 40 nM of ECO NP/siRNA was plated into each well and cell viability was assessed at 24, 48, and 72 h thereafter.

### 4.5. Efficacy of NP siRNA Delivery and Gene Silencing

Western blots were used to assess the silencing capabilities of ATM and DNApk-cs siRNA (40 nM) delivered with RGD-PEG NPs. Cells were incubated with NPs for 24 h after which the nanoparticles were removed, cells rinsed with PBS, and fresh media added. Cells were harvested at the indicated time points (48–120 h). Cell pellets were lysed on ice in RIPA buffer (Thermo Scientific, Rockford, IL, USA) supplemented with Complete mini EDTA-free protease inhibitor and PhosSTOP phosphatase inhibitor cocktails (Roche, Mannheim, Germany). Protein concentrations were determined via DC™ protein assay (Bio-Rad, Hercules, CA, USA). Protein (40 µg) was diluted 1:6 in 6X SDS protein loading buffer (Morganville Scientific, Morganville, NJ, USA), boiled at 95 °C for 5 min, run on a 4-20% tris-glycine gel and transferred using a Trans-Blot Turbo transfer system (Bio-Rad, Hercules, CA, USA). Membranes were blocked in 5% milk in PBS then incubated with primary antibodies overnight at 4 °C (1:1000 in 5% bovine serum albumin (BSA)). For protein detection membranes were incubated with near-infrared dye conjugated secondary antibodies (1:10,000) for 1 h at room temperature and imaged (LI-COR, Lincoln, NE, USA). The following antibodies were used: actin (M monoclonal [clone C4], MilliporeSigma-MAB1501R, Burlington, MA, USA), DNApk-cs (Rb polyclonal [Y393], abcam-ab32566), and ATM (Rb monoclonal [Y170], abcam-ab32420). 

### 4.6. Effect of NP Treatment and Radiation on DNA Damage Repair

The analyses of gH2AX foci before and after radiation were used to determine the effect of RGD-PEG ECO NP delivery of siRNA on DNA damage repair. Cells were seeded into glass chamber slides and allowed to attach overnight. NPs carrying 40 nM of negative control, ATM, DNApk-cs, or a combination of ATM and DNApk-cs siRNA (20 nM each) were added for 24 h then removed and replaced with fresh media. Cells were irradiated (2 Gy) 3 days after and collected at 6 and 24 h after radiation. Slides were fixed with 4% paraformaldehyde and stained for gH2AX (1:500 MilliporeSigma, 05–636). Slides were mounted with Prolong Gold Antifade with DAPI (ThermoFisher Scientific, Waltham, MA, USA), and imaged at 63x. The foci of 50 cells per group were counted.

### 4.7. Effect of NP Treatment and Radiation on Clonogenic Cell Survival

Tumor cells were seeded onto 60 mm dishes and allowed to attach overnight. NPs carrying either 40 nM of negative control, ATM, DNApk-cs, or a combination of ATM and DNApk-cs (20 nM each) were plated for 24 h after which NPs were removed and replaced with fresh media. Then, 3 days after, cells were seeded into 6-well plates (50–9600 cells/well) and irradiated (0.5–6 Gy) after they had attached. Cells were stained after 12–21 days using 0.5% crystal violet, colonies containing at least 50 cells were counted, and surviving fractions calculated. 

### 4.8. Effect of Radiation and Intratumoral Injection of RGD-PEG Labeled ECO NPs on Survival in an Orthotopic Model of GBM

The effect of intratumoral administration of RGD-PEG labeled ECO NPs carrying siRNA was evaluated with and without radiation in vivo. Athymic female nude mice (6-week old; NCr nu/nu; NCI Animal Production Program) were used. First, nylon guide screws with a 0.5 mm diameter channel were implanted into the skulls of mice 1 mm anterior and 2.5 mm lateral to the bregma, leaving the screw head exposed for repeated injections. Then, 3 days thereafter, U251 cells expressing luciferase (2.5 × 10^5^ in 2.5 µL) were injected through the guide screw to a depth of 3.5 mm at 1 µL/min. Bioluminescent imaging (BLI) was performed 3 days after tumor cell implantation at which time mice were randomized into experimental groups (n = 5). The following groups were evaluated alone and in combination with IR: control (vehicle of 7% ethanol in water), RGD-PEG ECO NPs loaded with negative control siRNA, and RGD-PEG ECO NPs loaded with siRNA targeting DNApk-cs. Because the nanoparticle material was suspended in ethanol, the control groups were injected with an equivalent concentration of ethanol (7% in water, 3 µL). Each NP injection (3 µL) was loaded with 10 pmol of siRNA. Mice received daily injections for 5 consecutive days. Mice receiving radiation were irradiated (2 Gy) on days 4, 5, and 6 for a total therapeutic dose of 6 Gy. On days where mice received IR, injections were performed at least 3 h before IR. Following the last irradiation mice were subjected to weekly BLI to monitor tumor growth and observed until the onset of neurologic symptoms. GraphPad Prism was used to generate Kaplan–Meier survival curves. All animal studies were conducted in accordance with the principles and procedures outlined in the NIH Guide for the Care and Use of Animals.

### 4.9. Statistical Analysis

GraphPad Prism 8 (Graphpad Software, La Jolla, CA, USA) was used for all analyses. For in vitro experiments, ANOVA with Tukey’s post-analysis was used to determine statistical significance (*p* < 0.5). For in vivo experiments, Kaplan–Meier curves were analyzed using a log-rank analysis.

## 5. Conclusions

In this study, we demonstrated the potential for the novel ECO nanomaterial to deliver single and combination siRNA to radiosensitize GBM in vitro and in vivo. In vitro, RGD and PEG labeled ECO nanoparticles were shown to effectively deliver a variety of siRNA, significantly inhibit protein expression, and impede DNA damage repair and clonogenicity in a GBM cell line (U251) and two glioma stem-like cell lines (NSC11 and GBMJ1). The delivery of siRNA simultaneously targeting complementary pathways of the DNA damage repair process, ATM and DNApk-cs, resulted in greater radiosensitization than either siRNA alone, proving the ECO nanocarrier to be a versatile vehicle for blocking the many pathways associated with tumor survival. More importantly for future application in the clinic, these nanoparticles resulted in no toxicity and limited silencing capabilities in normal astrocytes. In vivo, RGD-PEG labeled ECO nanoparticles effectively delivered siRNA targeting DNApk-cs upon intratumoral injection into orthotopic U251 xenografts, significantly increasing survival when combined with radiation compared to injection of nanoparticles alone. Overall, these data suggest the ECO nanomaterial has the potential to be a novel carrier of siRNA to more selectively target and radiosensitize tumor cells for the prolongation of survival in the GBM setting.

## Supplementary Materials

The following are available online at https://www.mdpi.com/2072-6694/12/11/3260/s1, Figure S1: Example western blot for (a) DNApk-cs and (b) ATM siRNA selection in U251 glioma cells using Lipofectamine transfection. Cells were transfected with 40 nM of each siRNA for 24 h. Samples were collected for western blot analysis at 48 h after plating siRNA. Hs_PRKDC_6 and Hs_ATM_5 were chosen for use in this study.
