# Supplementary material for: Improving Radiation Response in Glioblastoma Using ECO/siRNA Nanoparticles Targeting DNA Damage Repair
Source: Cancers (Basel). 2020 Nov 4;12(11):3260. doi: 10.3390/cancers12113260 (PMC7694254; doi:10.3390/cancers12113260)
Supplement: Supplementary file 1 [file cancers-12-03260-s001.pdf]

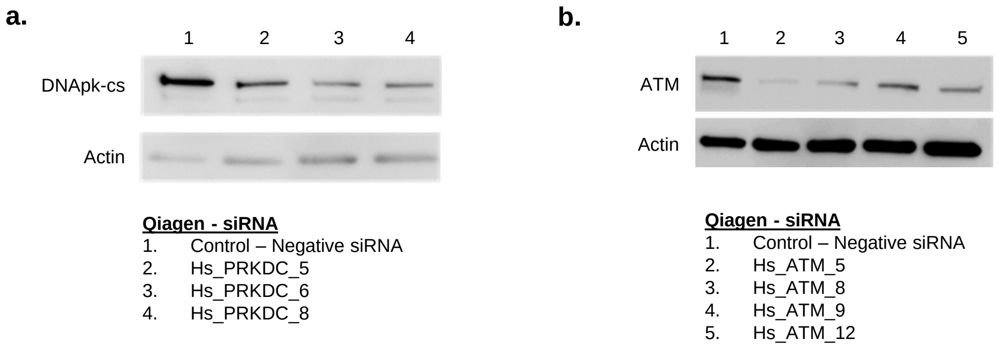

Supplemental Figure 1. Example western blot for **(a)** DNApk-cs and **(b)** ATM siRNA selection in U251 glioma cells using Lipofectamine transfection. Cells were transfected with 40 nM of each siRNA for 24 h. Samples were collected for western blot analysis at 48 h after plating siRNA. Hs_PRKDC_6 and Hs_ATM_5 were chosen for use in this study.
